# Regional Abnormality of Grey Matter in Schizophrenia: Effect from the Illness or Treatment?

**DOI:** 10.1371/journal.pone.0147204

**Published:** 2016-01-20

**Authors:** Ying Yue, Li Kong, Jijun Wang, Chunbo Li, Ling Tan, Hui Su, Yifeng Xu

**Affiliations:** 1 Shanghai Huangpu Second Mental Health Center, Shanghai, 200023, China; 2 College of Education, Shanghai Normal University, Shanghai, China; 3 Shanghai Key Laboratory of Psychotic Disorders, Shanghai Mental Health Center, Shanghai Jiao Tong University School of Medicine, Shanghai Jiao Tong University, Shanghai, 200030, China; 4 Bio-X Institutes, Key Laboratory for the Genetics of Developmental and Neuropsychiatric Disorders, Ministry of Education, Shanghai Jiao Tong University, Shanghai, 200030, China; 5 Department of Radiology, Ruijin Hospital affiliated with the School of Medicine, Shanghai Jiao Tong University, Shanghai, 200025, China; Institute of Psychology, Chinese Academy of Sciences, CHINA

## Abstract

Both schizophrenia and antipsychotic treatment are known to modulate brain morphology. However, it is difficult to establish whether observed structural brain abnormalities are due to disease or the effects of treatment. The aim of this study was to investigate the effects of illness and antipsychotic treatment on brain structures in antipsychotic-naïve first-episode schizophrenia based on a longitudinal short-term design. Twenty antipsychotic-naïve subjects with first-episode schizophrenia and twenty-four age- and sex-matched healthy controls underwent 3T MRI scans. Voxel-based morphometry (VBM) was used to examine the brain structural abnormality in patients compared to healthy controls. Nine patients were included in the follow-up examination after 8 weeks of treatment. Tensor-based morphometry (TBM) was used to identify longitudinal brain structural changes. We observed significantly reduced grey matter volume in the right superior temporal gyrus in antipsychotic-naïve patients with schizophrenia compared with healthy controls. After 8 weeks of treatment, patients showed significantly increased grey matter volume primarily in the bilateral prefrontal cortex, insula, right thalamus, left superior occipital cortex and the bilateral cerebellum. In addition, a greater enlargement of the prefrontal cortex is associated with the improvement in negative symptoms, and a more enlarged thalamus is associated with greater improvement in positive symptoms. Our results suggest the following: (1) the abnormality in the right superior temporal gyrus is present in the early stages of schizophrenia, possibly representing the core region related to schizophrenia; and (2) atypical antipsychotics could modulate brain morphology involving the thalamus, cortical grey matter and cerebellum. In addition, examination of the prefrontal cortex and thalamus might facilitate an efficient response to atypical antipsychotics in terms of symptom improvement.

## Introduction

Schizophrenia is a complex psychotic disorder characterized by significant brain abnormalities. Numerous imaging studies have revealed reduced grey matter volume in patients with schizophrenia involving multiple brain regions, such as the frontal cortex, temporal lobe, and insula [1]. Of these, the temporal lobe is most often affected [1]. In particular, the superior temporal gyrus has been reported as a common location of pathological brain aberrations in schizophrenia [2,3]. However, it is difficult to establish whether these structural abnormalities are caused by the disease or from the antipsychotic treatment. Studies of antipsychotic-naïve patients with first-episode schizophrenia will be helpful in identifying disease-related brain regions, which is crucial for understanding the pathophysiology of schizophrenia. The few studies in the literature that have investigated structural brain abnormalities in antipsychotic-naïve patients with first-episode schizophrenia and compared them to healthy controls have reported inconsistent results. Narayanaswamy et al. (2015) reported superior temporal gyrus deficits in antipsychotic-naïve schizophrenia patients based on whole brain analyses [4] whereas studies of some other regions of interest (ROI) studies additionally found reduced volume in the thalamus and cerebellum, in addition to reduced volume in the superior temporal cortex [5–7].

Accumulating evidence has indicated that antipsychotic medication might modulate brain morphology. For example, in a cross-sectional study comparing drug-free schizophrenia patients to schizophrenia patients treated with antipsychotics, Dazzan et al. (2005) demonstrated that increased thalamus volume was associated with atypical antipsychotics by comparing drug-free patients and patients with the use of antipsychotics [8]. Similarly, Deng et al. (2009) conducted a longitudinal study to investigate the effects of antipsychotics on brain structures in drug-naïve patients with schizophrenia after up to 8 weeks of treatment and found significantly increased grey matter volume in the thalamus, caudate nucleus, cortical regions (such as the frontal cortex, superior occipital cortex, and inferior parietal cortex) and cerebellum [9]. However, patients in this study were treated with mixed antipsychotics (typical and atypical). As Dazzan et al. (2005) noted, typical and atypical antipsychotics might affect brain structures differently due to their different pharmacological actions [8]. Therefore, studies of patients treated with only one type of antipsychotic could eliminate the confounding effects of mixed antipsychotics, and thus, identifying the brain structures related to the corresponding antipsychotic treatment.

In the present study, we first investigated whole brain structural changes by comparing antipsychotic-naïve patients with first-episode schizophrenia to healthy controls to identify the core regions related to schizophrenia. We further examined the effects of antipsychotics on brain structures after 8 weeks of atypical antipsychotic treatment. Few investigations to our knowledge have performed such a comprehensive study using antipsychotic-naïve patients to investigate disease-related changes in brain structure and then further performed a longitudinal study to examine the effects of atypical antipsychotics. This longitudinal study will be helpful in identifying small structural changes in the brain due to antipsychotic treatment in the early stage of disease.

## Method

### 2.1 Subjects

Twenty-five subjects diagnosed with first-episode schizophrenia were recruited for the present study. Two subjects did not complete scanning, and 3 subjects were excluded because of poor image quality. Therefore, 20 patients (10 males and 10 females) were ultimately included in the patient group, with a mean age of 24.45 years (SD = 5.51) and an average of 11.95 years of education (SD = 2.61). Patients received atypical antipsychotics at an average daily dose of 285 mg/day (SD = 108.94) of chlorpromazine equivalents (CPZ; [10]). Patients had not received antipsychotic medicine prior to scanning. All patients met ICD-10 criteria for schizophrenia and had received a Positive and Negative Syndrome Scale (PANSS) total score > = 60. Individuals with any history of the following were excluded: (1) serious somatic disorders; (2) central nervous system diseases; (3) alcohol or substance abuse; (4) extreme agitation; or (5) a restricted MRI examination. Ten of the 20 patients received a follow-up examination and brain scan after 8 weeks of treatment, and one patient did not complete the scanning. Therefore, 9 subjects (4 males and 5 females) with an average age of 23.00 (SD = 4.80) and an average of 11.22 years of education (SD = 2.64) were included in the follow-up study. The patients were treated with a second generation antipsychotic at an average of 300 mg/day (SD = 150.00) of CPZ equivalents (Clozapine n = 1, Quetiapine n = 6, Aripiprazole n = 1, Olanzapine n = 1). Psychopathological symptoms were rated using the PANSS [11]. Twenty-five healthy controls were also recruited, and one of these subjects was excluded because of poor image quality. Thus, 24 subjects were entered into the healthy group, which included 13 males and 11 females, having a mean age of 24.79 years (SD = 6.11) and an average of 13.17 years of education (SD = 2.16). None has any lifetime history of neurological or medical illness, head injury or substance abuse. Shanghai Mental Health Center- Institutional Review Board (SMHC-IRB) approved this study (Approval number: 2011-03R). All patients and controls provided written informed consent to participate in the study.

### 2.2 MRI acquisition

All subjects were scanned on a GE Sigma 3.0 T MR (GE Medical Systems, Milwaukee, Wisconsin) with T1-Weighted 3D magnetization with the following parameters: time to repetition (TR) = 7.8 ms; time to echo (TE) = 3.0 ms; flip angle = 7°; matrix = 256×256; voxel size = 1×1×1 mm^3^. Each scan was supervised by a trained technologist.

### 2.3 Image preprocessing and analysis

#### 2.3.1 Image preprocessing: Baseline analysis

All of the T1-weighed MRI images were processed using the voxel-based morphometry (VBM) toolbox in SPM8 (The Wellcome Department of Imaging Neuroscience, London; http://www.fil.ion.ucl.ac.uk/spm) with the default parameters on the Matlab 7.1 platform (The Mathworks, Natick, MA, USA). Detailed processing steps of VBM have been presented previously [12,13]. Briefly, all of the MRI images were first normalized and segmented into GM, white matter and cerebrospinal fluid. Subsequently, GM images were modulated and smoothed using default parameter settings.

#### 2.3.2 Image preprocessing: Longitudinal analysis

To investigate the volume changes in regional tissue after 8 weeks of treatment, MRI images were processed using tensor-based morphometry (TBM) [14], which was well confirmed by previous longitudinal studies [15,16]. The detailed steps were described in aforementioned studies but primarily included the following procedures: (1) follow-up scans were first registered to the baseline scans with a rigid transform; (2) a deformation tool (high-dimension deformation field) in SPM8 was used to detect the regional tissue changes over time and the amount of change (expansion or contraction) at each voxel was saved in the determination of the gradient of the deformation; (3) a customized grey matter template was created by averaging smoothed (8 mm), normalized, segmented grey matter images from all follow-up subjects; (4) follow-up grey matter images were normalized to the customized grey matter template (from step 3) and the normalization parameters were applied to the corresponding Jacobian determination (from step 2); (5) to create product images, normalized grey matter images were multiplied by the corresponding normalized Jacobian determination; and (6) finally, smoothed, modulated grey matter images and product images as follow-up and baseline grey matter images were entered for statistical analysis.

### 2.4 Statistical analysis

#### 2.4.1 Statistical analysis: Baseline study

Student’s t-test and chi-square tests using SPSS 17 (SPSS Inc., Chicago, IIIinois) were utilized to examine group differences in basic demographic characteristics and clinical data. Between-group differences in grey matter volume based on VBM analysis were analyzed using a t-test in SPM8, with age as a covariate. The results were obtained on a voxel-level height of threshold p<0.001, uncorrected, and a cluster-level extent threshold of 50 continuous voxels. The associations of grey matter volume in the distinct regions with PANSS and its subscores as well as untreated duration of illness were further performed using SPSS. The effect of untreated duration of illness on grey matter volume in the whole brain was also tested in the patients group by general linear models (GLM) in SPM.

#### 2.4.2 Statistical analysis: Longitudinal study

The demographic characteristics of the follow-up and non follow-up groups were performed using Student’s t-test and chi-square tests using SPSS. The normality of the grey matter volume of the sample was conducted with descriptive statistics in SPSS. A paired t-test in SPM was used to identify regional grey matter changes after 8 weeks of treatment. Multi-regression analysis was used to examine the associations between the changes in regional grey matter volume and PANSS scores. The results were observed with a voxel-level height of threshold p<0.05, family-wise error (FWE) corrected, and cluster size of 100. The change in PANSS from baseline to follow-up was examined using a t-test in SPSS 17. The effect of dosage of medication on grey matter volume was tested in the patients group by GLM in SPM.

## Results

### Baseline results

**Fig 1:**
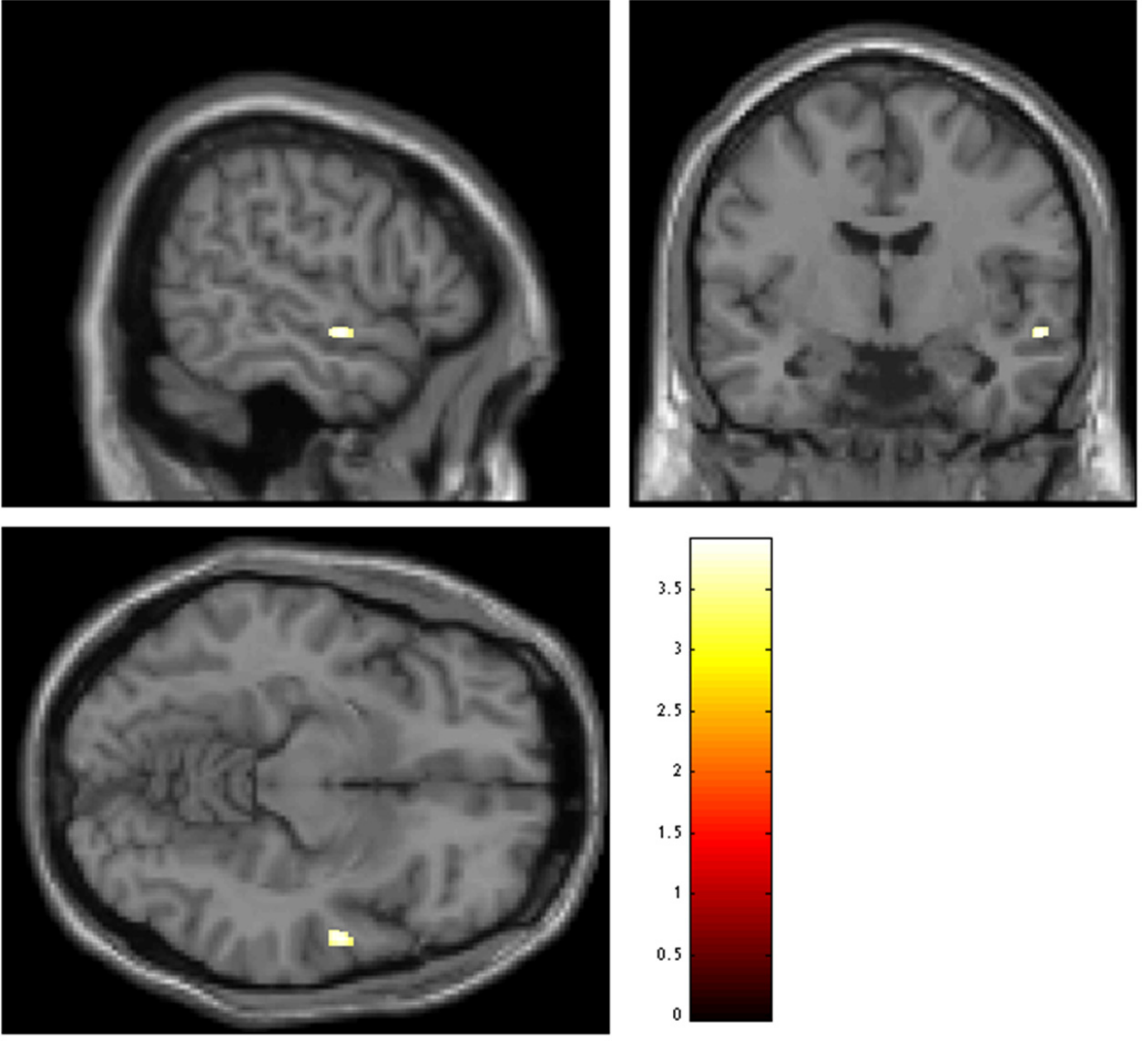
Regions of decreased grey matter volume at baseline in antipsychotic-naïve patients with schizophrenia compared to healthy controls. *P*<0.001, uncorrected, threshold = 50.

There were no significant differences between patients and healthy subjects in age, sex or education. The detailed information was described in Table 1(a). Compared to the healthy controls, patients exhibited significantly reduced grey matter volume in the right superior temporal gyrus (Fig 1, Table 2(a)). Further correlation analyses demonstrated that the right superior temporal gyrus volume was negatively associated with the PANSS total scores, positive and negative subscores (respectively, r = -0.061, p = 0.798; r = -0.094, p = 0.692; r = -0.252, p = 0.285), although none of them were statistically significant. We did not find significant association between untreated duration of illness and grey matter volume in the right superior temporal gyrus (the different region of patients with schizophrenia and healthy controls). We further performed the association in the whole brain, and we only found significant negative association between them in the left superior parietal gyrus, which was not survived after multiple correction.

**Table 1:** Demographic and clinical characteristics of (a) patients group and controls group and (b) follow-up group and non follow-up group.

(a)	Controls (n = 24) Mean (SD)	Patients (n = 20) Mean (SD)	*t* (df = 42)	*P* value
Age (years)	24.79 (6.11)	24.45 (5.51)	0.19	0.848
Education (years)	13.17 (2.16)	11.95 (2.61)	1.69	0.098
Sex (M/F)	13/11	10/10		0.783*
PANSS	n/a.	88.95 (14.86)		
Positive score	n/a.	21.25 (5.42)		
Negative score	n/a.	19.10 (6.26)		
General psychopathology	n/a.	48.60 (8.19)		
Duration of illness (years)	n/a.	1.91 (1.94)		
Medication (mg)	n/a.	285 (108.94)		
(b)	follow-up group (n = 9) Mean (SD)	non follow-up (n = 11) Mean (SD)	*t* (df = 18)	*P* value
Age (years)	23.00 (4.80)	25.64 (5.99)	-1.07	0.299
Education (years)	11.22 (2.64)	12.55 (2.54)	-1.14	0.270
Sex (M/F)	4/5	6/5		0.653*
Duration of illness (years)	1.68 (1.84)	2.10 (2.09)	-0.47	0.642
Medication (mg)	300 (150.00)	272 (64.67)	0.55	0.591

Data expressed as the means (SD); SD: standard deviation; df: degrees of freedom; PANSS: positive and negative syndrome scale; n/a.: not applicable;

*: χ^2^-test

**Table 2:** Anatomical structures showing significant GM abnormalities (a) decreased GM volume at baseline in patients with schizophrenia (n = 20) compared to healthy controls (n = 24); (b) increased GM volume from baseline to follow-up (8 weeks of treatment) in patients with schizophrenia (n = 9).

Anatomical structure	Cluster size (voxel)	*t* value	Peak Talairach coordinates x, y, z
(a)			
Right superior temporal gyrus	50	3.89	56, -6, -11
(b)			
Left superior frontal cortex, extending to right superior frontal cortex and left/right supplementary motor area	965	48.20	-2, 22, 40
Right middle frontal cortex	226	34.62	42, 10, 40
Left middle frontal cortex	348	28.78	-22, 22, 46
Left insula	451	27.41	-38, -14, 18
Left superior occipital cortex	147	25.47	-16, -78, 30
Right insula	518	25.11	38, -10, 16
Right cerebellum, extending to left cerebellum	304	23.91	2, -54, -50
Right thalamus	364	23.54	12, -24, 10

(a) Height threshold p<0.001, uncorrected, cluster size = 50;

(b) Height threshold p<0.05, corrected for family-wise error rate, cluster size = 100.

### Longitudinal analysis results

There were no significant difference between the follow-up group and non follow-up group in age, sex and education. The detailed information of the two sub-groups was described in the Table 1(b). Although the sample size of this longitudinal analysis is small, grey matter volume of the sample is approximately normally distributed (Shapiro-Wilk test p = 0.428).

**Fig 2:**
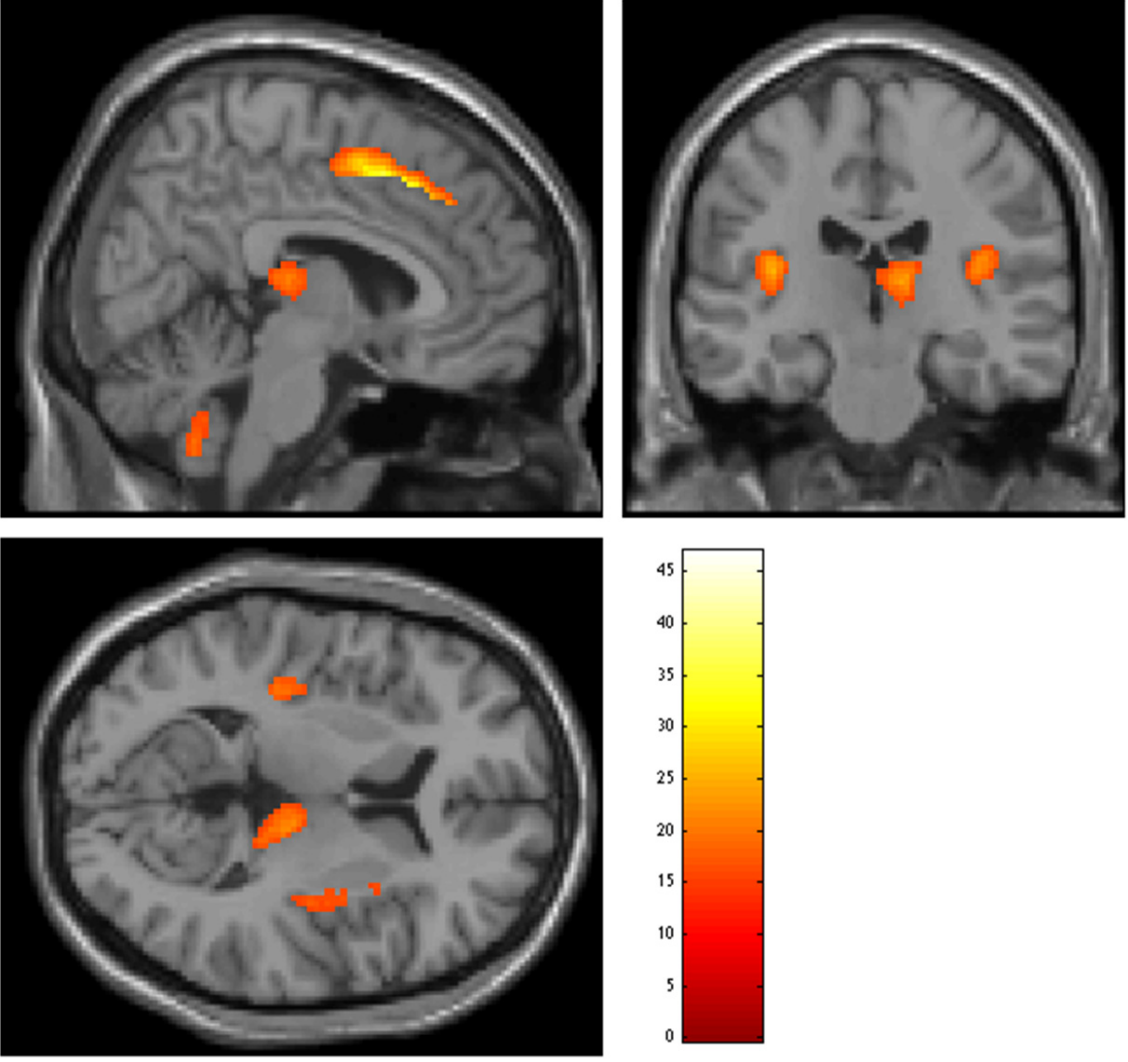
Regions of increased grey matter volume from baseline to follow-up in patients with schizophrenia.

After 8 weeks of treatment, pronounced grey matter increases were noted mainly in the prefrontal cortex (bilateral superior and middle frontal cortex), bilateral insula, left superior occipital cortex, left/right cerebellum and right thalamus (Fig 2, Table 2(b)). Significant decreases in the total scores of the PANSS and its subscores were also observed in the patient group (Table 3). Specifically, PANSS scores significantly decreased (from 94.33 (17.33) to 47.3 (13.01), t = 4.72, p<0.001), as did positive PANSS (from 21.56 (6.63) to 10.78 (2.91), t = 8.48, p<0.002), negative PANSS (from 21.00 (5.5) to 14.89 (5.57), t = 4.06, p<0.004) and general psychopathology (51.78 (10.22) to 33.44 (11.66), t = 7.72, p<0.001). In addition, a moderate correlation and a trend-wise level of significance was found for the association between increasing grey matter volume in the right middle frontal cortex and a greater improvement in PANSS total scores and its negative subscores (respectively, r = -0.7; p = 0.036; r = -0.635; p = 0.066); there was also a trend-wise level of significance for the association between increased grey matter volume in the right thalamus and a greater improvement of positive symptoms (r = -0.622, p = 0.074). The dosage of medication did not show significant effect on grey matter volume of the patients.

**Table 3:** Differences in PANSS scores from baseline to follow-up.

	Baseline Mean (SD)	Follow-up Mean (SD)	*t* (df = 8)	*p*
PANSS	94.33 (17.33)	59.11 (17.20)	8.48	0.000
Positive score	21.56 (6.63)	10.78 (2.91)	4.72	0.002
Negative score	21.00 (5.5)	14.89 (5.57)	4.06	0.004
General psychopathology	51.78 (10.22)	33.44 (11.66)	7.72	0.000

## Discussion

In this study, we first used VBM to investigate regional grey matter abnormalities in antipsychotic-naïve schizophrenia patients compared to healthy controls. We then examined the effect of antipsychotics on brain structures after 8 weeks of treatment in patients with schizophrenia. Our principal findings were that (1) decreased grey matter volume in the right superior temporal gyrus was observed in the early stage of the disease; and (2) after 8 weeks of antipsychotic treatment, patients showed significantly increased grey matter volume mainly in the bilateral prefrontal cortex, insula, right thalamus, left superior occipital cortex and the bilateral cerebellum. In addition, the grey matter volume in the prefrontal cortex and thalamus was associated with negative and positive symptoms, respectively.

Decreased grey matter volume in the right superior temporal gyrus was observed in the antipsychotic-naïve patients with schizophrenia relative to the healthy controls, which suggests an alteration in this region due to the disease. Our present results are consistent with those from a previous study investigating brain structural abnormality in antipsychotic-naïve patients with deficits in the superior temporal cortex [4]. Abnormal grey matter volume in the right superior temporal gyrus has often been reported in schizophrenia, including both first-episode [15,17,18] and chronic schizophrenia [19,20]. As reported in an earlier meta-analysis, the right superior temporal gyrus is one of the most consistently reported regions with reduced grey matter volume in schizophrenia [1]. The superior temporal gyrus has been found to be involved in auditory processing in addition to language functions and auditory memory [21,22]. The abnormality of grey matter in the superior temporal gyrus in patients with schizophrenia may be related to auditory and related higher cognitive functioning deficits in schizophrenia [4]. Our further correlation analysis also supports this view with the finding that the right superior temporal gyrus was negatively associated with the PANSS total score and the PANSS positive and negative scores, although these associations were not statistically significant (p>0.05). Additionally, we identified only one region in this study—the right superior temporal gyrus—that showed reduced grey matter volume in antipsychotic-naïve schizophrenia. This contrasts with previous findings which showed widespread grey matter abnormalities in both first-episode and chronic schizophrenia, suggesting that the structural abnormalities may have developed in the latter period. This view is also supported by our previous longitudinal study findings with progressive grey matter volume during the course of schizophrenia [15]. However, for the aforementioned results, it is difficult to exclude the effects of confounding factors such as medication and illness chronicity. Our present findings from our investigation of the grey matter abnormalities of antipsychotic-naïve patients at the early stage of the disease may indicate that the right superior temporal gyrus represents the core region of the pathological changes in schizophrenia.

Research has documented the ability of antipsychotic medication to produce brain volumetric changes even over a short period of time [23]. In the current study, patients with schizophrenia indeed demonstrated an increase in grey matter volume in multiple regions over 8 weeks of antipsychotic treatment. Similarly, other studies have also found that treatment consisting of atypical antipsychotic medications maintained or increased cortical grey matter volume in schizophrenia patients [8,24]. In this study, we observed increased volume in the thalamus of patients with schizophrenia after treatment, which is in line with previous reports showing an enlarged thalamic volume to be associated with use of atypical antipsychotics, using a region of interest technique [25,26] and voxel-based morphometry analysis [8]. Furthermore, our results demonstrate that the increase in thalamus volume after treatment was associated with the improvement of positive symptoms, a finding which supports the description of thalamic enlargement in association with improvements in positive symptoms after 4 weeks of treatment with atypical antipsychotics [26]. The thalamus, holding a key anatomic position in the brain, is part of the circuit that modulates perception, thinking and feeling and their integration in conscious experience [27]. Of note, then, is that thalamic volume appeared to be reduced in the patients with schizophrenia [28,29]. The thalamus has been proposed as a site participating in neural circuits mediating the clinical effects of antipsychotic drugs due to its role in the integration and coordination of brain activity [30]. Additional evidence has also been offered at the cellular and molecular levels, with increased expression of the Fos-like protein in the midline thalamic nuclei in response to antipsychotic drugs and an increased N-acetyl-aspartate (NAA) level in the thalamus in association with atypical antipsychotics [31]. Therefore, our present results might suggest that thalamus volume could be a candidate biomarker to evaluate the efficiency of the antipsychotic response in terms of improvement in the positive symptoms of patients.

Increased prefrontal volume was also found after treatment, which is consistent with the previous findings of increased cortical thickness in the prefrontal cortex over 8 weeks of atypical antipsychotic treatment in first-episode schizophrenia [32] and an enlarged prefrontal volume after 4 weeks of antipsychotic treatment for schizophrenia [33]. In addition, the increased prefrontal volume observed in the current study was found to be associated with the improvement of negative symptoms, which again nicely matched the finding by Goghari et al. (2013) showing increased prefrontal cortical thickness in association with the improvement of negative symptoms [32]. The prefrontal cortex, playing important roles in regulating, controlling and carrying out executive functions, has been observed with abnormal grey matter volume or thickness in many previous studies of schizophrenia [16,34–37]. Moreover, the abnormalities of prefrontal grey matter were often reported to be associated with negative symptoms in particular [38], although the exact mechanism of such a relationship remains unknown. A decrease in negative symptoms has been associated with dopamine release in the prefrontal cortex, which can be modulated by combined D2 and serotonin 5-HT2A receptor antagonism [39]. The increased grey matter volume in the prefrontal cortex that we found could be due to atypical induced neural plasticity and synaptic remodeling [39], and therefore, appeared to represent a beneficial increase from antipsychotic treatment.

The insula plays an important role in the regulating of emotion and cognition [40], and its abnormalities, such as reduced grey matter volume and decreased blood flow, have often been shown in patients with schizophrenia [41,42]. Another previous study [43] found decreased insular volume in antipsychotic-naïve patients with schizophrenia. However, in a later study from the same research group [44], this time involving patients with schizophrenia who were treated with antipsychotics, analyses showed equivalent insular grey matter volume in patients and healthy controls, suggesting insular volume increase after antipsychotic exposure. In addition, Pressler et al. (2005) showed a positive association between insular volume and dose-years (typical antipsychotics) [44], which indicated that the change in insular volume was related to antipsychotic effects. The association between abnormal insular volume and exposure to typical antipsychotics was also reported in Dazzan et al. (2005) [8], as they documented decreased insular volume in patients with typical antipsychotics relative to healthy controls. Despite inconsistent results regarding to the effects of typical antipsychotics on insular volume, the data appear to suggest that the insula could be a potential action site for antipsychotics. However, few studies have investigated the effects of atypical antipsychotics on the insular volume in patients with schizophrenia; so, this issue requires further examination.

The cerebellum is known to be involved with motor coordination as well as with aspects of cognitive functioning such as attention, working memory, and sensory discrimination [45]. As such, the cerebellum has been implicated in the pathophysiology of schizophrenia, with the cortico-thalamo-cerebellar circuit [46] receiving particular attention. Grey matter abnormality in this region has been demonstrated to be associated with antipsychotics. For example, Deng et al. (2009) found increased grey matter volume in the cerebellum in patients with schizophrenia after 8 weeks of atypical antipsychotic treatment [9]. Regarding this supposed association between the cerebellum and antipsychotics, Yegaheh-Doost et al. (2011) further provided molecular evidence in animal model, demonstrating that an atypical antipsychotics (clozapine) was associated with an increase in the expression of NR2C in the cerebellum relative to a typical antipsychotics (haloperidol). This finding suggests that clozapine may be superior to haloperidol in restoring a deficit in NR2C expression in the cerebellum [45].

Finally, we also observed an increase in grey matter volume of the superior occipital cortex associated with atypical antipsychotics. Prior research has also shown volume enlargement of the superior occipital cortex to be associated with antipsychotics [9], so this region could be another potential action site for antipsychotics. Based on our present findings, antipsychotics appear to primarily exert their therapeutic effects on disease-related regions. Because this study focused mainly on the early stages of schizophrenia, understanding whether and how these regions were affected by the disease process or exposure to antipsychotics during the course of schizophrenia still require long-term longitudinal study. The main contribution of the present study is that we addressed a core question how brain responds to early treatment, which contributes to the growing evidence that antipsychotic drugs may possibly have a role in modulating brain morphology during the early phase of schizophrenia, and aid to establish future treatment strategies in this disorder.

The present study also has some limitations. The modest sample size is the main limitation, although the study design was longitudinal. Additionally, this was a naturalistic study, meaning that the choice of medicine is clinician-led. In addition, the untreated duration of illness in the present study is a little long (mean duration of illness were 1.91 years). Although whether untreated duration of illness was associated with brain morphology in patients with schizophrenia was still equivalent [47] and our present results also demonstrated that the untreated duration of illness was not associated with the grey matter volume of the different region (right superior temporal gyrus) between schizophrenia and healthy controls, it is important to note that it is difficult to completely exclude the contributing effect of untreated duration of illness on brain morphology in patients with schizophrenia. Future longitudinal studies with larger samples and with shorter untreated duration of illness will be needed to confirm the current findings. However, despite these limitations, this study had a number of strengths. We used antipsychotic-naïve first-episode schizophrenia patients to minimize the effects of illness duration and prior exposure to antipsychotic medication on grey matter volume. Moreover, we longitudinally assessed patients from baseline to eight weeks after the beginning of treatment. Finally, we comprehensively evaluated the whole brain grey matter structure structural changes related to antipsychotic treatment, unlike previous research which focused only on particular regions of interest [32].

Therefore, based on our findings, we suggest that (1) the right superior temporal gyrus may represent the core region of pathological change in schizophrenia; and (2) the effects of atypical antipsychotics could involve multiple regions, and examination of increased grey matter volume in the prefrontal cortex and thalamus may be particularly effective in evaluating the efficiency of response to exposure to atypical antipsychotics in the improvement of schizophrenia symptoms.
